# Dynamics of Freedom: Negotiating Constraints

**DOI:** 10.1007/s12124-024-09859-3

**Published:** 2024-08-02

**Authors:** Jonas Tellefsen Hejlesen

**Affiliations:** Department of Communication and Psychology, Aalborg University, Aalborg, Denmark

**Keywords:** Agency, Self, Desire, Sociogenesis, Reflexivity, Subjective freedom

## Abstract

In this paper, I grapple with the question of why we, at times, experience ourselves as not free. In doing so I outline a crude theory of agency (and our experience of ourselves as free) as a dynamic process happening in irreversible time. In attempting to answer this question, I define agency as the ability to pursue our desires, and I claim that we experience ourselves as free as long as we can do this – with the caveat that the ability to reason is a necessary criterion. I show that agency is a sociocultural development that manifests as the ability to reason gradually develops through social interaction during infancy and into adulthood. Crucially, I point out that reason is a double-edged sword: It allows us to question our actions and desires and whether they are worth pursuing, which is what elevates us to agentic beings. However, it also allows us to alienate ourselves from our actions and desires, and thus rob ourselves of our experience of freedom. Lastly, I show how our subjective freedom is lost and gained in a constant process, generated by a reflexive-relating-to ourselves. As we act, we continually encounter constraints (physical and psychological) that bar us from acting upon our desires. This compels us to reflect on our actions and desires, and so, our feeling of freedom evaporates. However, through a retrospective forgetting, or reconstruction, of the constraints we encounter, we may regain our experience of being free.

## What does it mean to be free?

What does it mean to be free? This deceptively simple question, it seems, has no singular answer. Within philosophical discussions on free will, there is no clear agreement on how to define the concept. While incompatibilists (whether libertarians or free will sceptics) most commonly define free will as ‘the ability to do otherwise’, a common compatibilist definition is ‘the ability to act according to one’s wants’ – or simply stated, to pursue your desires.^1^

The primary disagreement seems to revolve around the stringency regarding the criteria needed for free will to be present. According to the compatibilist definition, I need only to have been able to act differently *in theory*, while the incompatibilist definition requires the ability to act differently *in practice*. Suppose I want to sell one of my kidneys (and let’s say, for simplicity’s sake, that I reside in a country where this is allowed, Iran). I have the freedom to do this if I want to. Also, I have the freedom to refrain from doing it if I do not want to. From a compatibilist perspective, this is all that is required: I am free if I could have done otherwise *had I wanted to do otherwise –* that is, if there are no constraints that bar me from bringing my want to fruition. However, from an incompatibilist view, this is insufficient. Mere possibility is not enough; I must also have been able to want to do otherwise in the first place.^2^

Stepping away from the philosophical discussions on free will and into folk psychological understandings of free will, there seems to be substantial convergence between the compatibilist conception of free will and dominant folk psychological understanding. Monroe and Malle (2010) finds the common-sense definition of free will to be something along the lines of “(…) a choice that follows one’s desires and is not internally or externally constrained.” (p. 215) – a finding that is corroborated by Lam (2021). This definition consists of three key ingredients (*choice*,* following one’s desires*, and *the absence of external and internal constraints*) that might be compressed into the phrase ‘the ability to pursue what you want.’^3^

For the sake of clarity, I will use the term *agency* to refer to the compatibilist and common-sense conception of free will, the ability to pursue your wants. When I talk of freedom or someone being free, this is what I mean. On the other hand, I take the concept of free will to mean what incompatibilists typically mean: the ability to do otherwise – to be able not just to do what you want, but also, as Schopenhauer (1841/2009) famously remarked, to want what you want (or rather to not want what you want, and want what you do not want).^4^

In this terminology, the subject of this paper is not free will but agency – the phenomenology of freedom, rather than the ontology of freedom. I take this perspective, as our experience of ourselves as free seems to be bound up with agency rather than free will per se. Central to my argument is the proposition that we experience ourselves as free as long as we can pursue our wants, and only when we cannot do so do we find that our experience of ourselves as free breaks down.

### According to What Desire?

With agency defined as the ability to pursue your wants, it is obvious that intentional action is central – acting on one’s desires necessitates this ability. However, this is not enough. Rather, it seems that first when a person can reason for themselves do they fit the conditions of being an agent. We find this reflected in our legal practices. For example, are children, people who are (severely) intellectually disabled or insane not considered fully agentic – rather they have limited rights and responsibilities. And in the case of all three of these groups, what is lacking is exactly the ability to reason (independently).

It is not just in our social and legal practices that this idea shows itself, but it is also present in the philosophical disucssions on the topic. In Harry Frankfurt’s (1971*)* paper, *Freedom of the Will and the Concept of a Person*, he makes a distinction between *first-order* (I want to do *x*) and *second-order desires* (I want to want to do *x*) (see also Indius’ concept of *metaintentionality*; Indius, 2022; Indius, 2023). On the back of this distinction, Frankfurt (1971) argues that only beings capable of second-order desires can be free; and only when second and first-order desires are in alignment are we free – that is, when we want something, and we want to want it. Infants – as well as other beings without the ability to reason – do not possess second-order desires. If I cannot reason independently, I am unlikely to ask – and especially answer – the question ‘What do I want to want?’ Rather, I would be what Frankfurt (1971) calls a *‘wanton’* (p. 11), a being that simply follows their immediate desire(s) without considering whether they want to have the desire itself (and thus, whether they should act upon it). To such a being, the very presence of the desire itself is what justifies it – because I want to do this, I do it.

A different, albeit related distinction, is expressed by Watson (1975). He argues that “[t]he problem of free action arises because what one desires may not be what one values, and what one most values may not be what one is finally moved to get.” (Watson, 1975, p. 209). From this conceptualisation of agency, the ability to reason is central once again. Beings with no or limited reasoning abilities cannot (properly) consider whether they value what they desire – they simply desire it.

Both views run into significant problems, however. As Watson (1975) asks of Frankfurt’s view, “(…) what prevents wantonness with regard to one’s higher-order volitions[?]” (p. 218). To prevent this, one would have to ask not just ‘Do I want to have this desire?’, but also ‘Do I want to want to have this desire?’ – and so one might continue. As Frankfurt (1971) himself admits, an infinite regress of higher-order desires can be imagined (p. 16) – and thus, at some point, we must simply take a ‘leap of faith.’ And to Watson, on the other hand, we might pose the epistemic question, how do I know what I value? Do I merely value what I say I value (given that I am not lying)? Or can I be mistaken – can I believe that I value one thing but truly value another? One might ask, is the very acting upon the desire not *the* supreme value judgment? Is the willingness to bring about the desire not precisely an expression of its value (to the subject)?

Critiques aside, Frankfurt (1971) and Watson (1975) both – despite their differences – express the view that while being free means that I can do what I want to do, it does not mean I do everything I want to do. If I do everything I want to do, I am not free. I am a slave of my desires. And in order not to be a slave to my desires, the ability to reason is required. I have to possess the ability to do a ‘reflexive movement’, relating to and (at times) intervening on my desires – as is also expressed by Heider (1958, p. 133ff). Following this, agency consists not just in acting according to our desires, but in acting according to the right desires, in knowing which desires to act upon.

## The Sociocultural Development of Agency

So far, I have defined agency as the ability to pursue your wants. Furthermore, I have argued that, while the ability to act intentionally is central, the ability to reason (and particularly reflexivity or metacognition) is indispensable. In the following sections, I outline the sociocultural development of these two key ‘traits’ – intentionality and reasoning – and thus, of agency.

### Persistent Imitation: The Basis for Intentional Action

It is no secret that infants ‘act’ as soon as they are born – they laugh and cry, they kick and reach and so on. However, it would be a stretch to argue that these actions are, at this point already, intentional. Rather, we might reasonably believe that these behaviours are merely preconscious and reflexive. Therefore, I will contend with the fundamental question of how the infant moves from (preconscious) reflexive behaviour to (conscious) intentional action in the first place.

To understand this, J. M. Baldwin’s (1895/1906) concept of *persistent imitation* – which he argues is the child’s first exhibition of volition (p. 349) – is crucial. To put it simply, in *simple imitation* – the ‘counterpart’ of persistent imitation –, the infant tries its best to imitate the intended action, but when it is unsuccessful, it does not attempt to do better – rather it merely repeats the same action. In persistent imitation, however, the same action is not merely repeated – rather, the infant furthers its attempts, through repetition, trying again and again until it is successful (Baldwin, 1895/1906, p. 126; 359f). And when the imitation is successful, effort (generally) subsides, and the action becomes habit (Baldwin, 1895/1906, p. 358f).

As an important aside, it is not just in infancy that persistent imitation takes place. Rather, it continues throughout our lives – as development is a never-ending process that continues throughout our lives (Baltes et al., 1999; Overton, 2010), and persistent imitation is a driving force behind development itself.

It seems that infants aged 18 months can distinguish between intentional and unintentional actions (Meltzoff, 1995), while they do not do so at 12 months of age (Behne et al., 2005).^6^ Furthermore, at 14 months of age, infants show clear signs of understanding – and executing – goal-directed actions. When imitating the action of an adult, they only imitate the exact action, “(…) if they consider it to be the most rational alternative” (Gergely et al., 2002) – if that is not the case, they choose the simplest or most intuitive means to obtain the intended goal. Even more importantly, when imitating unsuccessful goal-directed actions, infants aged 18 months (unlike infants aged 12 months) do not simply imitate the executed (i.e. failed) action – rather they imitate the intended action (Bellagamba & Tomasello, 1999).

Following this, we see how, in the process of persistent imitation, the infant goes beyond mere copying of action. As it attempts to copy an action that it is incapable of copying, it must strain itself to execute the intended action. Therefore, what begins as a preconscious, reflex-based behaviour (repeating an observed action) becomes a proto-intentional (i.e. conscious and goal-directed) action (continuously repeating an action *until it matches what was observed*). If the goal is attained on the first attempt (i.e. through simple imitation), the behaviour remains pre-conscious. However, if the first attempt is unsuccessful, but continuous efforts are made and the action is (at some point) executed successfully, this may be the initial push that ‘provokes’ the gradual move toward intentional action.

Note that the crucial thing here is the move from unsuccessful attempt(s) to successful attempt. Bear in mind that I do not argue that this move is itself intentional (i.e. due to the infant being able to actively and consciously change its way of acting) – this would be circular. Rather, the move may be merely incidental^7^ or made possible due to some biological development (e.g. sensorimotor), for example. However, when the infant persists in an action that it is unable to execute, we might assume that some frustration is building, and once it succeeds, the frustration disappears, resulting in a feeling of pleasure or satisfaction. Through repeated experiences of this type, the infant may become conscious of its behaviour due to this ‘feedback mechanism’ which may lay the groundwork for intentional action.

### Caregivers and Their Infants: The Co-construction of Agency

With the development of the ‘proto-agency’ of intentional action, we shift our attention to the co-construction of agency with the socio-cultural development of reason being *the* vital process.

In his essay *Saying ‘No’*, Donald W. Winnicott (1960/2016) comments on a conversation between several mothers regarding the degree to which they grant their infants freedom and responsibility throughout infancy and into early childhood. From the conversation, Winnicott outlines three phases concerning agency expressed by the mothers (Winnicott, 1960/2016, p. 35).^8^ In the first phase, the infant has no agency, and the caregiver takes full responsibility for it and its actions. The caregiver does not tell the infant what to do or what not to do; rather, they manipulate the world around the infant, so that it can roam (relatively) free (Winnicott, 1960, p. 40). The implicit message of the caregiver is ‘You think you are free, but really, I have already decided for you what you can do – but you do not know this yet. *You think you are free because I allow you to.*’

In the second phase, the caregiver begins to support the development of agency in the infant. As Winnicott states, this phase begins “when instead of saying ‘No’ to the world around, the mother says ‘No’ to her child” (Winnicott, 1960/2016, p. 35–40). At this point, a sort of *derivative agency* or *agency by proxy* is instantiated, as the caregiver tells the infant what to do, but leaves it up to the infant to act in accordance with their demands.^9^ The infant has some agency to act, but it is derived from the caregiver who places external constraints and thus imposes themselves and their views on the infant (Winnicott, 1960/2016, p. 35). In this phase, the caregiver seems to tell the child ‘You can do what you want – as long as it is also what I want you to (or at least not what I do not want you to). *You are as free as I allow you to be.*’

Slowly, the third phase – which is ‘the dawn of reason’ – emerges. The caregiver moves from the unexplained prohibition – ‘No!’ – to reasoning with the infant – ‘No, because…’ (Winnicott, 1960/2016, p. 35). At this point, one might talk about *distributed agency*; the caregiver reasons on behalf of the infant, such that it gradually begins to understand the reasoning behind prohibitions (and permissions). The caregiver implies ‘You can do what you want as long as you choose correctly. *You are free to act how reason requires*.’

From infancy, through childhood and into adolescence, the weight of the distribution changes such that what started as parent-reasoning-for-child becomes parent-reasoning-with-child, child-reasoning-with-parent, child-reasoning-with-symbolic-parent and so on. The shared reasoning becomes (more and more) superfluous as the child manages to internalise the reasoning processes until they can reason independently (presupposing normal development) – which marks the final phase of (full) agency.^10^

## The Double Bind of Reflexivity

Earlier, I wrote that our experience of ourselves as free seems to be bound up with agency. I stated that we experience ourselves as free as long as we can pursue our wants and that it is only when we harbour conflicting desires that we cannot bring in alignment that our experience of ourselves as free breaks down.^11^ However, one might very well suppose that beings who lack reason (e.g. infants and non-human animals) experience themselves as free. After all, they do what they want and not what they do not want.

If the experiential aspect is crucial, as I argue it is, how come I do not perceive e.g. infants and non-human animals as agentic? The answer is to be found in the fact that they cannot, as already stated, pose the question ‘Is this what I want?’ As they cannot ask this question, they cannot experience a conflict of desires – which I argue is the source of our unfreedom. As they cannot experience themselves as unfree, I would contend that they cannot truly experience themselves as free either – the reflexive movement is necessary for this experience. Thus, our reflexivity, the very thing that hands us our freedom, is also exactly what might rob us of it.

Due to our reflexivity, we find ourselves in a constant bind of negotiating our agency – losing it (while never really losing it) and gaining it back (while never really gaining it). Take the example of dieting. Let us begin from a point where I simply eat what I want. I act implicitly or pre-reflexively; I do what I want without even having to consider what I want. Suddenly, something happens which brings me to consider my eating habits – a movement of reflexivity which introduces some doubt and creates intrapsychic conflict. Perhaps summer is on the way, and I would like to be skinny for summer. Rarely do we simply say to ourselves, ‘Okay! I want to be skinny, so I will stop eating unhealthily’ – or if we do, we are (as a rule) confronted with the reality of the ambiguity of our desires sooner rather than later. More commonly, what happens in this instance is that our psyche becomes a battleground for conflicting desires: ‘I want to be skinny, but at the same time I really like eating sugary or fatty foods.’

At this point, I enter a sort of deadlock: I have conflicting desires, and I do not know how to bring the conflict to an end, as I do not know what I want for certain. Hopefully, at some point, a second movement of reflexivity happens, and I manage to break the deadlock. I might think ‘To Hell with beauty standards. I savour the pleasure of eating well more than being skinny’ – and thus, I can simply return to eating what I want. I no longer have to reflect on what I want; I need not consider it. I am firmly decided. Or perhaps I think ‘If I eat this chocolate bar, I will feel a short-lived momentary pleasure, but afterwards, I will feel guilty and regret it… It is more important for me to look nice!’ – and thus, I relieve myself of the tension-filled state by introducing the constraint: ‘You cannot eat unhealthily.’ – once again, I can act pre-reflexively; I know that I do not want unhealthy foods, so it is no longer an option for me. Or I create certain rules to bypass it: ‘You cannot eat unhealthy food… *except* on Fridays… at parties’ and so on.^12^

Provokingly, I believe that the very thing we might consider the epitome of unfreedom – being ‘a slave to one’s desires’ (spontaneous action, pre-reflexive living) – is the very source from which our freedom springs. I believe, our freedom resides in being a wanton that can be not a wanton – in being able to relate to our desires when confronted with the necessity of doing so.

Let’s continue with the previous example of dieting – perhaps the penultimate example of the exertion of agency in contemporary Western society. The paradox at the core of this ‘practice’ is that it functions exactly due to the insertion of a prohibition – and the same is the case for practices such as vows of silence, chastity and so on.^13^ In these cases, one binds oneself to the action – which is the purpose of the vow. Once the prohibition has been introduced, one need no longer reflect on what to do. Even more so, it practically becomes psychologically impossible to do otherwise. And paradoxically, it is exactly in these instances where we feel truly free; when we need not consider what we want, but we simply act out of the deepest conviction that we *know (*and do) what we want.^14^

### Agency and the Self

At this point, the reader might wonder, how come we ever experience ourselves as not free? Why is it that we, as Frankfurt (1971) outlines, may find ourselves in doubt as to what we want? Why is this ‘ambiguity of desire’ something that appears? Why do we, at times, find that we are at war with ourselves?

In the Western world, the notion of the self has long been central to our understanding of the person (see e.g. Taylor, 1989). Traditionally, the self has been – and to a large part still is – conceived as ‘the essence’ of a person – the source of personal identity, the thing that makes a person *that person* (across time and space). In the notion of the self, we find the conception that there is some fundamental, immutable property pertaining to one’s being, i.e. what it means to be me. Following this, the self is often conceived as ‘monological’ rather than dialogical (see Linell, 2009) – that is, as speaking with one voice, as internally consistent. And so, when conflict is present, we suspect that this is due to a lack of understanding (see e.g. Plato, 1992, 45f; Plato, 1997, p. 137f) rather than a fundamental problem – while I do not know for sure what want, a firm answer to this question does exist.

However, this conception of the self has been challenged in numerous ways (e.g. James, 1890/1983; Suzuki, 1934/1991; Markus & Kitayama, 1991; Hermans, 2001; Gergen, 2011). As well-worn phrases like being ‘not myself’, ‘beside myself’, or ‘losing myself’ reveal, we are (or at least feel like we are), while being ‘ourselves’, at times something other than ourselves, which of course begs the question, how can I be not-myself if this is the very definition of what it means to be me?

Many of the different dissenters of the traditional conception of the self suggest (albeit in different ways) that there is no (singular) self – that the person is not an integrated whole, but more like a collection of fragmented parts hanging together loosely. Through such reconceptualisations, a move is made from unity to multiplicity, from harmony to cacophony – i.e. from self as ‘concordant’ to self as ‘discordant.’ And based on this conception of the self, we might assert “(…) there is no Self as a substantive agent of psychic life” (Žižek, 2012, p. 129).

Once we make this shift – from the conception of the self as an integrated whole to a collection of fragmented parts – the problem of agency changes markedly; and we may understand why the earlier critique of Frankfurt’s (1971) hierarchical theory of agency does not reveal a flaw in the theory, rather, it reveals a ‘flaw’ in human desire itself. From such a perspective, our psyche is characterised by ‘polyvocality’ (see e.g. Linell, 2009, 119f; Wertsch, 1991, p. 53–56) rather than being univocal – and so, ambiguity, contradiction and conflict are to be expected.

## The Dynamics of Freedom

If it is the case that our psyche itself is multiplicitous, we will inevitably be confronted with the ambiguity of our desires, and there will be times when we are ‘at war with ourselves.’ In that case, freedom will remain a process to maintain rather than a state to obtain. We may manage to broker a truce, to negotiate a cease-fire, alas, it is always fickle and fragile. Sooner or later – and as a rule, sooner rather than later – conflict will resume. Ambiguity will return. And we will, once again, find ourselves suspended between contradictory demands. And so, the key question however remains how this temporary ‘cease-fire’ might be attained.^15^

**Fig. 1:**
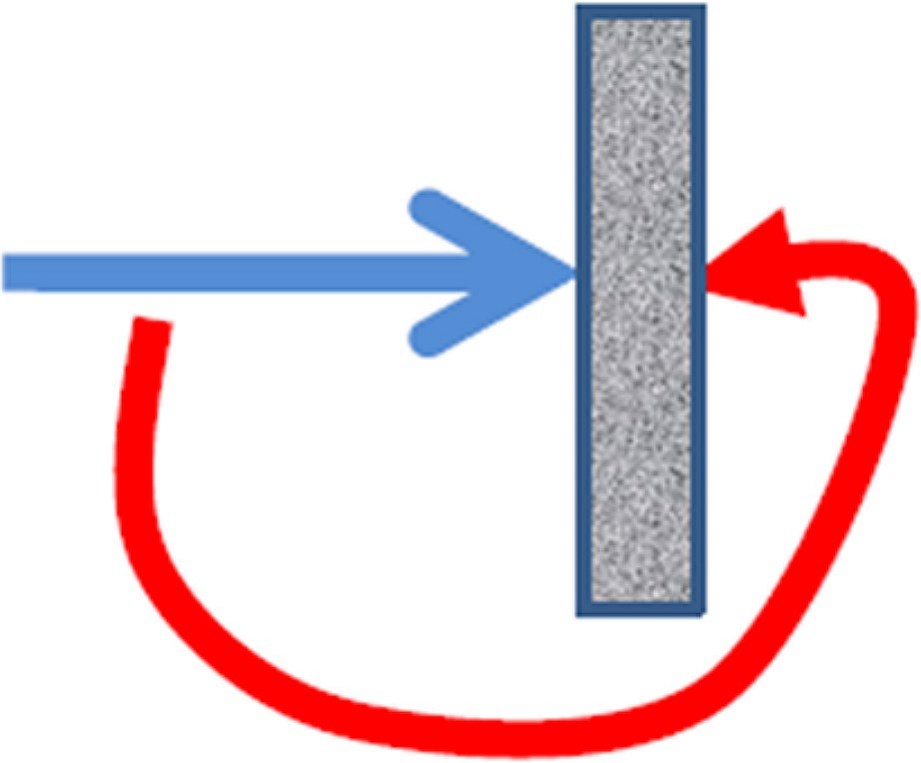
The *Gegenstand*-structure which consists of *a direction* (represented by the blue arrow) – in this instance in terms of desire and intention; *a border* that resists it (represented by the rectangle); and a negotiation between the two (represented by the red arrow)

In moving toward an answer to this question, it might be fruitful to introduce the concept of *Gegenstand* (see e.g. Valsiner, 2014a, b, 2020). Gegenstand is a barrier or a force that – as is the literal meaning of the word – *stands* (stand) *against (*Gegen) our actions (see figure 1). It is “(…) an object that, when acted upon, resists some aspects of the action” (Valsiner, 2014b, p. 289). And as such, we might conceptualise the dissolution of agency as an encounter with the border in the Gegenstand – something that resists our acting on our desire or which prevents the very knowing which desire to act upon.

As we are confronted with the border, some counteraction – a ‘recalibration’ of our efforts – is necessary. If we do not recalibrate, we will simply be confronted with the border over and over again. If we adjust our efforts, however, we might ‘traverse’ it. This can be done in several ways. Most commonly, the border appears on the level of action, and we will generally reassess our methods and try other means (‘I have tried to get x by doing this, but it does not work, so I will try this instead’).

If, for example, we take the case of a drug addict who tries to kick his drug habit. First, the person might simply try not to use the drug – without doing anything in particular to keep them from acting on the desire to use when it appears. When this does not work after repeated attempts (as is often the case), the person might change their efforts. They might get rid of the drug such that they will first need to obtain it once the desire appears. If this readjustment is successful and the person does not act on the desire to use the drug, then the feeling of freedom may reappear.

However, is it probably as – if not more – likely that this attempt is futile as well, resulting in the feeling of unfreedom persisting. Then, they might change their efforts so that it is harder for them to obtain the drug in the first place (e.g. deleting contacts from their phone). Thus, they no longer only have to refrain from obtaining the drug and then from using it, but now they must also refrain from recovering the contact information of a drug dealer. If this is unsuccessful as well, they might continue readjusting again and again, until their efforts are successful. Another thing that might also happen is that they come to question the sincerity of their wish – ‘I say I wish to quit using the drug, but I keep using it – so do I really wish to quit?’ – thus making themselves conscious of the ambiguity of their desires.

If the person cannot give a firm answer to this question, the border moves from the level of action (‘I want to pursue this goal, but how do I attain it?’) to the level of desire (‘I am not sure what goal I want to pursue’). In this case, things become a tad more complicated – and rather than a readjustment of how one is to pursue the desire, it is the desire itself that is reassessed. Thus, we are confronted with the impossible question ‘What should I do?’ – a question that we can only know the answer to retrospectively (i.e. once we have done what we should have done, or after having done the wrong thing and we realise what we should not have done). And here, following the example above, the person might conclude that the preceding unsuccessful attempts are evidence that they do not truly want to stop using – and thus, they give up the desire itself (‘I no longer want to stop using’) which may reintroduce the feeling of being free.

### The Art of Forgetting

The astute reader will have noticed that I stated that one’s feeling of freedom *may* be rescued when one has overcome the border. Why only *may?* How come the feeling of unfreedom persists at times? What is the process that leads to the feeling of freedom returning after the overcoming of the border? Here we come to the importance of forgetting.

**Fig. 2:**
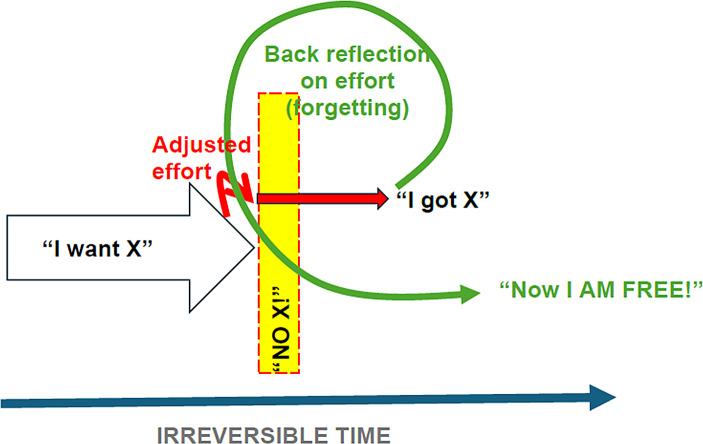
Model outlining the dynamics of agency and the process of regaining agency (as it happens in irreversible time; see e.g. Valsiner, 2011). I desire *x*, but I am encountered by something that bars me from realising this desire. Through adjusting my efforts, I attain *x* (or the desire itself changes, *x* becomes *y*). And finally, I retrospectively reflect on the adjusted effort, forgetting (or at the very least reconstructing) it

For agency forgetting is of central importance. If I remain fixated on the fact that I encountered some constraint and the considerable efforts this resulted in, then this ‘fixation’ might lead me to obsess over how my actions were constrained and how I was not free to do what I wanted. However, if I manage to forget – or at least convince myself that the encountered border in the Gegenstand and the subsequent adjustment(s) were inconsequential – then I will feel free. For example, after a ‘recalibration’ on the level of desire, I might reason ‘Maybe I thought I wanted x, but, really, I wanted y all along’ – thus, ‘purposively forgetting’ that I was practically forced to give up my desire. Or, on the level of action, I must ‘purposively forget’ e.g. that I had to go to extra lengths (i.e., in the case of the drug addict, get rid of the drugs, erase contacts and so on), and shift my focus from the efforts to the fact that I managed to execute action – that my pursuit was successful (see figure 2).

However, this ‘purposive forgetting’ is not always the mechanism through which agency is rescued. When we look closer, we see that it is also possible to go to the other extreme – that is making the border the centre of our attention, rather than forgetting or downplaying it. In these cases, the border becomes reconstructed as a testament to our freedom rather than an obstruction to it. For example, I might want to go for a run, but it is below the freezing point, and thus, I would also prefer to stay inside where it is warm and cosy. If I end up committing to my initial action, I may very well stress the presence of the terrible weather. The fact that I went for a run *despite* the blistering cold shows my dedication and willpower – it only further proves my freedom as I did what I wanted despite the circumstances that may very well have discouraged me from doing so.

Thus, agency is rescued through a ‘retrospective affirmation’ – either by reconstruction of, or a retrospective forgetting of the efforts required after the encounter with the border in the Gegenstand.

### The Temporality of Freedom

In the previous sections, the temporal dimension of agency becomes abundantly clear. We see how agency is not a property of the human agent, nor a state to be obtained by it. Rather it is a continual process – it is a doing and a becoming. It is a way of relating to the world and ourselves across time. Thus, agency can only be understood temporally – as it appears and disappears.

As Valsiner (2018) asserts, borrowing a concept from Bergson, living is “(…) unfolding in *irreversible time*” (p. 506) – and this is something that we must take into account. When it comes to agency, this means understanding it as a dynamic process and investigating these dynamics temporally – as outlined above. When we see agency not as something that is or is not there when a person acts, but as something that is continually being negotiated even after the action itself, the importance of the temporal aspect cannot be overstated.

## General Conclusion

In this paper, I have dealt with what we might call ‘the problem of agency’ – that is, why we experience ourselves as free at times while feeling unfree at other times. I have briefly outlined the development of agency, the criteria necessary for the experience of freedom to be present and why we find it breaking down at times. Through this, I unveil the ambiguity of our desires, outlining the dynamics of freedom, coming to an understanding of agency, not as an ability to be attained or a property that we possess, but as a perpetually recurring process –as something that must be continuously maintained.

To summarise the crucial points outlined and argued in this paper:

- Agency (which entails having desires and intentions, the ability to act on them, and finally the ability to reflect on and assess one’s desires and subsequent actions and adjust accordingly) is (while being an inherent ability) developed socioculturally – that is, through interaction with, first and foremost, the caregiver, but also the environment in general.
- Agency can be construed as developing in four different phases: *no agency*; *derivative agency*; *distributed agency*; and finally, *(full) agency*. Crucial for the final stage is the ability to reason which is what makes the person capable of a reflexive relating-to their desires and actions, creating the possibility for a recalibration of one’s action trajectory.
- I have argued that the experience of ourselves as free breaks down when we cannot pursue our desires. Following this, I outline a view of the person not as an integrated whole, but rather more like a collection of fragmented parts. From this perspective, we are not concordant but discordant selves. Our desires are not singular or harmonious; they are multiplicitous and often incongruent, antagonistic even – and this ambiguity is a fundamental part of our psyche.
- Furthermore, I have asserted that our freedom consists in us being wantons who have the ability to not be wantons when this is necessary. When we are confronted with the ambiguity of our desires, it is our reflexiveness that allows us to break the deadlock that has ensued. However, it is also the reflexivity itself that creates the experience of ourselves as unfree. Thus, our freedom and our unfreedom springs from the same source – our reflexivity.
- Following this latter point, we find ourselves in a constant process (generated by our reflexive-relating-to ourselves) of losing and gaining our freedom through continuous negotiation of different conflicting desires.

## Data Availability

No datasets were generated or analysed during the current study.

## Footnotes

1 I say *pursue* here to stress that this does not entail that you necessarily *get* what you want. You can do what you want (act upon your desire) without being successful (realising your desire). If I want to play in the NBA, I may be free to *pursue* that want, but whether I get to play in the NBA, is not (solely) up to me.

2 As an aside, there is agreement, across the compatibilist/incompatibilist-distinction, that wanting precedes acting. If I do something, it must be because there is some desire to do the thing – viz. there is no desireless action.

3 The desire-condition is explicitly contained: it is *what you want* that is pursued. The choice-condition is also there albeit implicitly: pursuing what you want rather than what you do not want. Lastly, the absence of external and internal constraint-condition is also implicitly there: it is what you want – not what others want (external), nor what you might be forced to want psychologically (internal).

4 Here it becomes evident how there is always some degree of internal constraint. However, when speaking of internal constraint, what it important – as we will come to see – is *reasons-responsiveness*; that is, that the person can consider and be persuaded by reason.

6 It seems both chimpanzees and capuchin monkeys also have the ability to distinguish between intentional and non-intentional actions (Premack & Woodruff, 1978; Call et al., 2004; Phillips et al., 2009; Call & Tomasello, 2008).

7 Imagine, for example, an infant that rocks forward to grasp an object just outside its reach. It can not reach it, but it rocks again and again, building momentum which finally allows it to grasp the object. For this to happen, the infant need not intend to ‘build momentum’ or be able to reason ‘If I keep rocking, I might build enough momentum…’.

8 Despite Winnicott’s use of the term ‘stage’, I will use the term phase (which is still not optimal), as the development should be conceived as gradual and continuous rather than sudden comprehensive stage-like changes.

9 Following up on what was outlined in the previous paragraph, it seems plausible that this builds upon what I deemed ‘the groundwork for intentional action.’ Once the infant can be conscious of its actions, and it begins to receive intentions from the outside, it is pushed further toward intentionality proper. We might suspect that the infant slowly learns to act on intentions as they are mandated (and at times forced upon it), and then, gradually it learns to set intentions to act upon itself.

10 In this slow but steady developmental process, external constraints are loosened in parallel with the psychological and cognitive maturation that takes place which leads to a loosening of the internal constraints and leaves the person as a ‘reasons-responsive’ being capable of expressing agency. These two processes – the developmental aspects (psychological and cognitive maturation) and the sociocultural aspect (recognition of agency and loosening of external constraints) – feed into each other, bringing about the gradual development of agency.

11 The idea that we experience an ‘intrapsychic conflict’ (that we seek to resolve) when we hold inconsistent ideas that we cannot integrate is most popularly expressed by Festinger (1957) in his theory of cognitive dissonance (see also Festinger et al., 1956). Naturally, we might question whether this phenomenon is a fundamental attribute of the human psyche or whether it might be socioculturally contingent, as ‘our’ thinking (in ‘the West’) is generally grounded in classical logic where contradiction is (per the ‘law’ of non-contradiction) the most blatant expression of flawed thinking – rather than dialectical thinking where contradiction is understood as fundamental.

12 This following process (and the role of reflexivity itself) can be understood through Jaan Valsiner’s theory of *semiotic meditation*. What is happening in the example given is the use of signs to regulate actions and the concurrent creation of *sign hierarchies* – i.e. use of signs to regulate other signs (enhancing, inhibiting, terminating). For more, see e.g. Valsiner, 1999; Branco & Valsiner, 2010; Valsiner, 2021. Here the key issue is of course why one is swayed by one rather than the other – what it is, in the example above, that brings one to diet or not to diet.

13 Interestingly, this maps on to the first phase of agency which, as mentioned, begins to develop with the introduction of prohibition, i.e. ‘the no of the father.’

14 One might protest that the insertion of a prohibition is necessary for free actions in the case of dieting, but in the case I choose not to diet, no prohibition is introduced – and so, following the logic, of the two, only dieting is a free action, while not dieting is not. However, I would retort that even in the case of not-dieting, we have the introduction of prohibition – namely, the prohibition of prohibition itself. When it comes to food, I am allowed to do whatever I want – except for creating limitations and restrictions on what I eat, that is.

15 A similar idea is comprehensively outlined by Lewin (1951) with his Force Field Analysis and the related concept of *quasi-stationary* equilibrium.
